# Hepatotoxicity Studies in the Progeny of Pregnant Dams Treated With Methimazole, Monocrotophos and Lead Acetate

**DOI:** 10.4103/0971-6580.75868

**Published:** 2011

**Authors:** K. Vanisthasree, A. Gopala Reddy, B. Kalakumar, C. Haritha, B. Anilkumar

**Affiliations:** Department of Pharmacology and Toxicology, College of Veterinary Science, Rajendranagar, Hyderabad - 500 030, India

**Keywords:** Hepatotoxicity, lead acetate, methimazole, monocrotophos, rats

## Abstract

An experimental study was conducted to evaluate the hepatotoxic effects in the progeny of dams treated with methimazole, monocrotophos (MCP) and lead acetate. Female pregnant albino rats of *Wistar kyoto* strain were divided into five groups and treated as follows, from day 3 of pregnancy till weaning of pups on postnatal day (PND) 21. Group 1 served as sham control, group 2 received methimazole 0.02% in drinking water, group 3 received MCP (0.3 mg/kg orally), group 4 received lead acetate at 0.2% in drinking water and group 5 received MCP + lead acetate. Thyroid hormone profile was recorded on 14 ^th^day of gestation in dams. Eight pups from each group were euthanized on PND 21 and 90, and liver tissues were collected for analysis. Thiobarbituric acid reactive substances (TBARS), protein carbonyls and reduced glutathione (GSH) of liver were studied on PND 21 and 90, while the activities of Na ^+^/K^+^ATPase and Mg ^2+^ATPase in the liver were studied on PND 90. T_3_, T_4_, GSH, Na^+^/K^+^ATPase and Mg^2+^ATPase were significantly (*P*<0.05) decreased, while TBARS and protein carbonyls were significantly (*P* < 0.05) increased in all the test groups as compared to group 1. From this study, it is concluded that both MCP and lead acetate have a possible influence on thyroid gland of dams as the thyroid profile was altered significantly and the hepatotoxic effects were comparable to those induced by methimazole.

## INTRODUCTION

Long-term low-level exposure to lead may result in reduced free T_4_concentration.[1] There is a strong correlation between maternal and umbilical cord blood lead levels, indicating the transfer of lead from mother to fetus.[2] Several data suggest that for lead, the main toxic event is prenatal exposure.[3] In addition, lead levels in breast milk also increase proportionately with the lead level in maternal blood, posing an additional risk to the neonates/nursing infants.[4] Zaidi *et al*.,[5] reported that upon exposure to the dust and liquid formulation of endosulfan, monocrotophos (MCP), parathion and fenvalerate, levels of TSH were elevated and T_4_ levels were decreased as compared to the values of control group. Therefore, based on these earlier findings, it is hypothesized that organophosphate an d lead compounds could affect maternal thyroid hormone availability for the fetus and therefore affect the development of the neonates. Further, studies on the combination of these compounds (MCP + lead acetate), a natural feature in environmental pollution, have rarely been attempted. Also, there is a need to establish a link, if any, between the exposure of mothers to these pollutants and altered thyroid homeostasis in both the dams and fetuses, and the onset of a syndrome of toxicological manifestations in the neonates. Hence, this study was taken up to evaluate the hepatotoxic effects of these pollutants individually and in combination on pups whose mothers were treated from 3^rd^ day of gestation till the weaning of the pups.

## MATERIALS AND METHODS

Female albino rats of *Wistar kyoto* strain, weighing about 200-250 g, were procured from National Institute of Nutrition, Hyderabad. The animals were housed in solid bottom polypropylene cages. Animals were given commercial standard mash feed and provided water *ad libitum*. Experiment was conducted according to the guidelines of Institutional Animal Ethics Committee. Five groups comprising eight dams in each were mated and the progeny raised were maintained from postnatal day (PND) 1 to 90. The dams were maintained as per the following schedule from day 3 of pregnancy till the weaning of pups (PND 21).

Group 1: Sham
Group 2: Methimazole 0.02% in drinking water
Group 3: MCP at 0.3 mg/kg orally
Group 4: Lead acetate at 0.2% in drinking water
Group 5: Combination of MCP + lead acetate as detailed in groups 3 and 4

Only thyroid profile was studied in dams, while the progeny were subjected to the liver tissue analysis. Blood samples were collected from the dams on 14^th^ day of gestation and sera samples were separated to estimate thyroid hormone profile by radioimmunoassay. Eight pups from each group were euthanized on PND 21 and 90 for analysis. Thiobarbituric acid reactive substances (TBARS),[6] protein carbonyls[7] and reduced glutathione (GSH)[8] of liver were studied on PND 21 and 90, while the activities of Na^+^ /K^+^ATPase[9] and Mg^2+^ ATPase[10] in the liver were studied on PND 90. Protein estimation was done by the method of Lowry *et al*.,[11] The data were analyzed by one-way analysis of variance (ANOVA) using statistical package for social sciences (SPSS) version 15. Differences between means were tested using Duncan’s multiple comparison test and significance was set at *P*<0.05.

## RESULTS AND DISCUSSION

The mean concentrations of T_3_ (ng/dl) and T_4_ (*μ*g/dl) were significantly (*P*<0.05) reduced, while that of TSH (*μ*U/ml) was significantly (*P*<0.05) increased in all the treated groups of dams as compared to that of control group 1 (158.68±21.46, 3.83±0.14 and 0.06±0.02, respectively). Thyroid hormone profile was estimated in order to establish the influence of MCP and lead acetate on the functioning of thyroid. It is well documented that acute exposure to high levels of persistent organic pollutants and heavy metals can affect human health including thyroid function[12] by significantly decreasing the concentration of T_3_ and T_4_ and significantly increasing TSH. Methimazole is a known anti-thyroid drug, while MCP and lead acetate are strong inducers of oxidative stress. Hence, it is hypothesized that the free radicals generated in dams following exposure to these chemicals might have induced dysfunctioning of thyroid.

The concentration of TBARS [*μ*M malondialdehyde (MDA)/mg protein] in liver revealed a significant (*P*<0.05) rise in progeny rats of all the test groups on 21^st^ and 90^th^ days as compared to group 1 rats (0.12 0.02 and 0.18±0.03, respectively). The concentration of protein carbonyls (nM/100 mg protein) in liver recorded a significant (*P*<0.05) increase in progeny of all the test groups on 21^st^ and 90^th^ days as compared to group 1 rats (1.42±0.18 and 1.78±0.07, respectively) [Table 1]. Lead is known to produce oxidative damage in the liver tissue by enhancing peroxidation of membrane lipids, a deleterious process solely carried out by free radicals.[13] Organophosphates have been reported to increase TBARS concentration (an indicator of lipid peroxidation) on sub-chronic exposure.[14] Free radicals cause peroxidation of lipids, resulting in the formation of aldehydes such as TBARS, while oxidation of proteins results in the formation of carbonyls. In this study, concentration of TBARS and protein carbonyls was increased in liver in the progeny pups of treatment groups, suggesting ongoing oxidative stress. This finding is in accordance with an earlier report by Elvira *et al*.,[15] which shows similar findings on oxidative stress following prenatal exposure to lead.

The concentration of GSH (mM/g tissue) in liver revealed a significant (*P*<0.05) reduction in the progeny rats of all the test groups on 21^st^ and 90^th^ days as compared to group 1 rats (4.23±0.39 and 4.04±0.17, respectively) [Table 1]. Lead also has a very high affinity for the sulfhydryl groups of GSH, which has implications for the maintenance of the thiol-disulfide balance in the cell.[16] Hepatic and extrahepatic GSH depletion and glutathione-S-transferase (GST) inhibition were observed in different tissues of albino rats that were given a single oral dose of MCP.[17] In the present study, concentration of GSH was reduced significantly in the treated groups in liver as compared to pups of control group. The results of oxidative stress on the biomarkers revealed a correlation between the action of methimazole and toxic test compounds (MCP and lead acetate). As the stress parameters of methimazole can be attributed directly to the anti-thyroid effects of the drug, the simulating actions of both MCP and lead acetate can be correlated to anti-thyroid actions in fetuses and neonates, besides direct free radical generating ability of these test chemicals, which enter into the fetus through maternal circulation and to the neonates through milk while nursing.

There was a significant (*P*<0.05) decrease in the activity (*μ*M of Pi released/mg protein/30 min) Na^+^ /K^+^ ATPase and Mg^2+^ ATPase in the liver of all the groups (2-5) of test progeny as compared to that in group 1 (16.56±1.73 and 10.75±0.86, respectively) [Table 1]. The action of membrane-bound enzymes depends on the cellular membrane integrity; an altered integrity of membrane following derangement of membrane lipids due to lipid peroxidation may affect the functioning of membrane ATPases, subsequently leading to ionic imbalances in the cells. This is evident from the results of the present investigation, which revealed increased lipid and protein peroxidation.

In conclusion, the study revealed that the hepatotoxic effects recorded in the progeny of dams treated with MCP and lead acetate were comparable to those of the progeny of dams administered with methimazole, a proven anti-thyroid drug. Therefore, it may be possible that both MCP and lead acetate might have a possible influence on the thyroid gland of dams as the thyroid hormone levels, which play an important role in early prenatal life in the proper development of fetus, were significantly altered in dams and were comparable among the treatment groups.

**Table 1:** Results of oxidative stress and ATPases in liver of progeny rats

Group	TBARS (uM of MDA/mg protein)		Protein carbonyls (nM/100 mg protein)		GSH (mM/g tissue)		Na/K^+^ ATPase (*μ*M of Pi released/mg protein/30 min)	Mg^2+^ (*μ*M of Pi released protein/30 min)
	PND 21	PND 90	PND 21	PND 90	PND 21	PND 90	PND 90	PND 90
Control	0.12±0.02^aA^	0.18±0.03^aA^	1.42±0.18^aA^	1.78±0.07^aA^	4.23±0.39^aA^	4.04±0.17^aA^	16.56±1.73^a^	10.75±0.86^a^
Methimazole	0.19±0.03^bA^	0.29±0.02^bA^	2.76±0.26^bA^	3.33±0.19^cA^	1.46±0.37^cA^	1.37±0.21^cA^	5.32±0.71^c^	5.69±0.18^c^
MCP	0.21±0.01^bA^	0.31±0.04^bA^	2.31±0.32^bA^	2.52±0.11^bA^	3.31±0.21^bA^	2.87±0.19^bA^	9.20±1.62^b^	6.67±0.36^b^
Lead acetate	0.22±0.02^bA^	0.34±0.01^bA^	2.53±0.41^bA^	2.70±0.19^bA^	2.90±0.29^bA^	2.41±0.15^bA^	12.54±1.41^b^	8.99±0.40^b^
MCP+ lead acetate	0.27±0.02^cA^	0.44±0.02^cA^	2.81±0.31^bA^	3.43±0.15^cA^	1.53±0.22^cA^	1.42±0.36^cA^	5.46±0.44^c^	3.26±0.62^d^

Values are Mean±SE (*n* = 8); one-way ANOVA (SPSS). Means with different alphabets as superscripts differ significantly (*P*<0.05); capital alphabets indicate horizontal comparison between time intervals and small alphabets indicate vertical comparison among groups; TBARS, Thiobarbituric acid reactive substances (TBARS); GSH, reduced glutathione; MCP, monocrotophos
